# COVID-19 Outcomes in a US Cohort of Persons Living with HIV (PLWH)

**DOI:** 10.3390/reports5040041

**Published:** 2022-10-09

**Authors:** Amanda Blair Spence, Sameer Desale, Jennifer Lee, Princy Kumar, Xu Huang, Stanley Evan Cooper, Stephen Fernandez, Seble G. Kassaye

**Affiliations:** 1 Division of Infectious Diseases, Georgetown University Medical Center, Washington, DC 20007, USA; 2 MedStar Health Research Institute, Hyattsville, MD 20782, USA; 3 Department of Medicine, Georgetown University Medical Center, Washington, DC 20007, USA

**Keywords:** HIV, COVID-19, SARS-CoV-2, complications, outcomes

## Abstract

Reported coronavirus disease 2019 (COVID-19) outcomes in persons living with HIV (PLWH) vary across cohorts. We examined clinical characteristics and outcomes of PLWH with COVID-19 compared with a matched HIV-seronegative cohort in a mid-Atlantic US healthcare system. Multivariate logistic regression was used to explore factors associated with hospitalization and death/mechanical ventilation among PLWH. Among 281 PLWH with COVID-19, the mean age was 51.5 (SD 12.74) years, 63% were male, 86% were Black, and 87% had a HIV viral load <200 copies/mL. Overall, 47% of PLWH versus 24% (*p* < 0.001) of matched HIV-seronegative individuals were hospitalized. Rates of COVID-19 associated cardiovascular and thrombotic events, AKI, and infections were similar between PLWH and HIV-seronegative individuals. Overall mortality was 6% (*n* = 18/281) in PLWH versus 3% (*n* = 33/1124) HIV-seronegative, *p* < 0.0001. Among admitted patients, mortality was 14% (*n* = 18/132) for PLWH and 13% (*n* = 33/269) for HIV-seronegative, *p* = 0.75. Among PLWH, hospitalization associated with older age aOR 1.04 (95% CI 1.01, 1.06), Medicaid insurance aOR 2.61 (95% CI 1.39, 4.97) and multimorbidity aOR 2.98 (95% CI 1.72, 5.23). Death/mechanical ventilation associated with older age aOR 1.06 (95% CI 1.01, 1.11), Medicaid insurance aOR 3.6 (95% CI 1.36, 9.74), and multimorbidity aOR 4.4 (95% CI 1.55, 15.9) in adjusted analyses. PLWH were hospitalized more frequently than the HIV-seronegative group and had a higher overall mortality rate, but once hospitalized had similar mortality rates. Older age, multimorbidity and insurance status associated with more severe outcomes among PLWH suggesting the importance of targeted interventions to mitigate the effects of modifiable inequities.

## 1. Introduction

Data are mixed regarding the severity and clinical outcomes of coronavirus disease 2019 (COVID-19) among persons living with HIV (PLWH) and there are reports of both similar and worse clinical outcomes among different populations of PLWH co-infected with SARS-CoV-2. Early studies indicated a similar COVID-19 illness severity among PLWH and HIV-seronegative counterparts, but many of these reports were case series and/or had no matched controls [1–11]. There are also reports of higher rates of death and/or more severe disease among PLWH internationally and domestically [11–15]. Much of the literature describing COVID-19 in PLWH is limited to case series, single-center studies, includes mostly individuals on antiretroviral therapy (ART), and/or does not have matched control cohorts [1,3,9,16]. However, even among population-based or registry studies, there is variability in reported outcomes, with some reporting increased mortality risk or hospitalizations and others not noting this association [14,15,17,18]. Thus, additional data across are needed to describe the impact of COVID-19 on PLWH.

There are consistent reports of higher disease severity or worse outcomes among PLWH with COVID-19 who have medical comorbidities such as diabetes, cardiovascular disease, obesity and/or chronic lung disease [19,20]. COVID-19-associated complications such as acute kidney injury (AKI), cardiac events/myocardial injury, thrombosis, and stroke are well reported in the literature, and risk factors for these events include pre-existing disease and/or other medical comorbidities [21–33]. However, the occurrence of these COVID-associated events have not yet been described in PLWH, a population with high prevalence of medical comorbidities including diabetes, obesity, hypertension, and dyslipidemia [34–37]. These COVID-19-associated events can account for significant morbidity, mortality, and healthcare expenditure among persons affected by COVID-19. Thus, an assessment of COVID-19 related outcomes and infection associated events is needed among PLWH.

We conducted a retrospective analysis of all PLWH diagnosed with COVID-19 seen in the MedStar Healthcare system. The MedStar Healthcare system is the largest healthcare provider in the Maryland and Washington, DC area that includes 10 hospitals and provides ambulatory care services in the hospitals and at free standing sites in the surrounding communities. These facilities serve urban, suburban, and rural populations [38]. The District of Columbia and Maryland have some of the highest rates of HIV in the country with 2360.8 and 652.9 diagnoses per 100,000, respectively [39]. Our sampling provides a representative sample of PLWH in the Mid-Atlantic region who sought clinical care. This allows for a detailed analysis of clinical characteristics of PLWH compared to a matched cohort as well as examination of COVID-19 associated events as we sought to characterize clinical characteristics of PLWH who were infected with COVID-19 as well as outcomes as compared to the HIV-seronegative.

## 2. Methods

### Cohort Population and Data Sources:

All persons with a diagnosis of HIV, determined either via International Classification of Diseases (ICD-10) coding or laboratory testing with a diagnosis of COVID-19, who received care in the Medstar Healthcare system were included in this analysis. Individuals were considered to have a diagnosis of COVID-19 if an ICD-10 diagnosis code for COVID-19 or positive laboratory testing for SARS-CoV-2 by polymerase chain reaction (PCR) was documented between January 2020 and November 2020. In addition, an age- and sex/gender-frequency-matched control group of HIV-seronegative individuals with a diagnosis of COVID-19 was generated in a 1:4 ratio for comparison. There were no required standardized hospital protocols for hospital admission or COVID treatment and patient care decisions were based on the discretion of individual care providers.

### Variable Selection:

Demographic and clinical data were extracted from the Electronic Health Data Warehouse (MedStar Analytics Platform). Clinical data for this analysis included laboratory testing, medications/therapeutics, comorbid diagnoses as determined by ICD-10 coding, socio-demographics, oxygen requirements, and hospital length of stay. Individuals were considered to have multimorbidity (e.g., the co-occurrence of two or more chronic conditions) [40] if they had more than one of the following diagnoses: cardiovascular disease, obesity, diabetes, chronic renal disease, malignancy, or transplant. To determine accuracy of ICD-10 coding diagnoses, 25% of participants with each reported comorbid diagnosis were verified by manual chart review and the overall accuracy rate was 79% which is similar to other reports of discharge coding accuracy in the literature [41]. For those hospitalized, all laboratory data during the hospital admission were extracted. HIV viral load and CD4 + T lymphocyte count were obtained from the time most proximal to admission and/or COVID-19 diagnosis and viral loads were obtained until November 2020.

### Analytic Plan:

Descriptive statistics were used to describe cohort characteristics. Chi-square or Fisher’s exact tests were used for categorical variables and t-tests or Kruskal–Wallis tests were used for continuous variables to determine group differences [42] We examined differences in PLWH diagnosed with COVID-19 and PLWH without a diagnosis of COVID-19 as well as PLWH and HIV-seronegative individuals with a diagnosis of COVID-19. Univariate and multivariate logistic regression was used to explore factors associated with hospitalization and incident death/mechanical ventilation requirement among PLWH. Variables with a *p*-value < 0.05 in the univariate analysis or selected based on known effect on SAR-CoV-2 outcomes were included in the multivariate analysis [43]. Data utilized for these analyses are included in the manuscript text and tables. All analyses were completed in R 4.0.0.

## 3. Results

**Figure 1.**
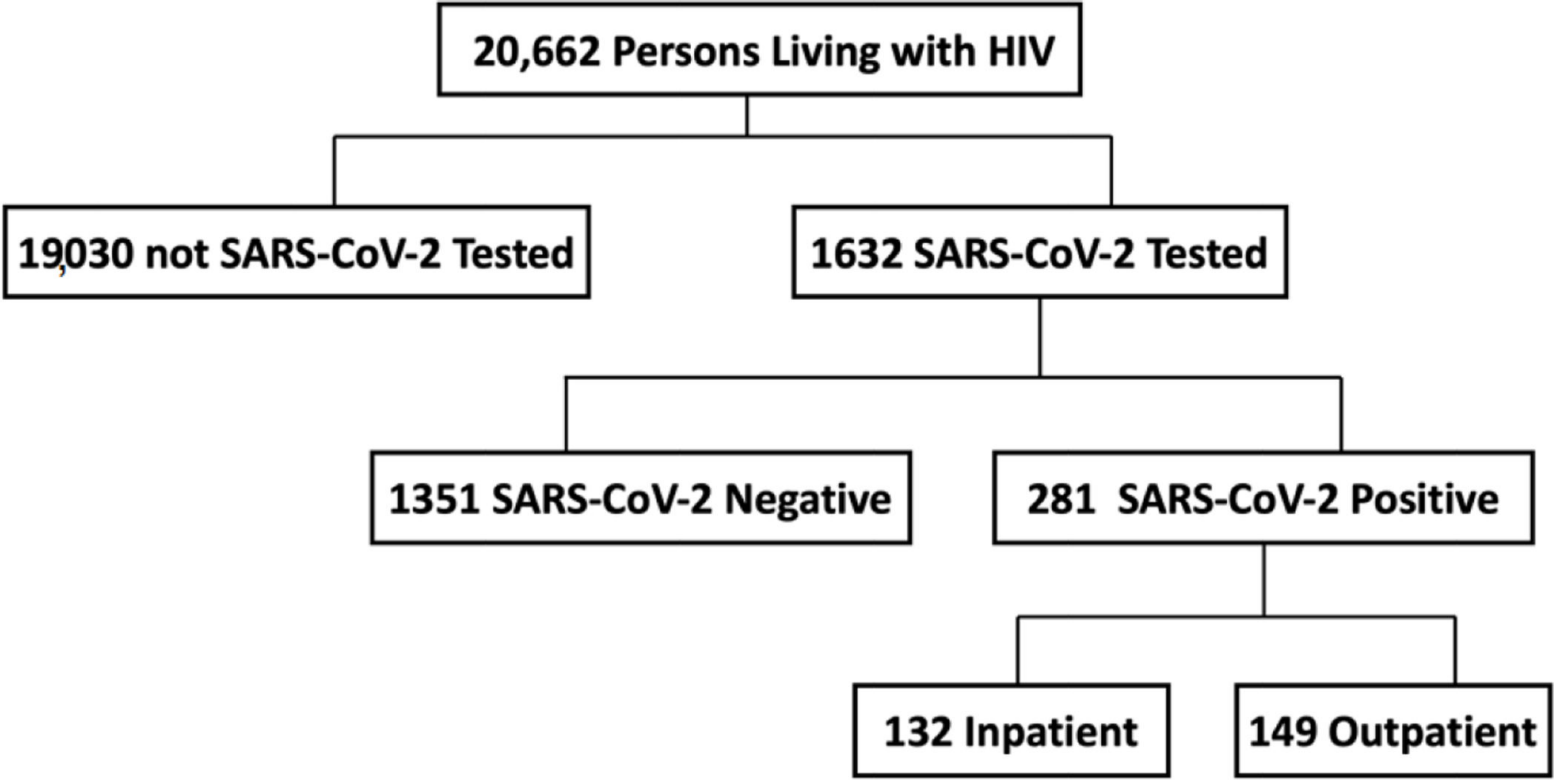
PLWH seen in the MedStar healthcare system with and without SARS-CoV-2 Testing. Flow chart of persons living with HIV seen in the MedStar Healthcare system. Abbreviations: HIV, Human Immunodeficiency Virus; PLWH, Persons Living with HIV; SARS-CoV-2, Severe Acute Respiratory Syndrome Coronavirus 2.

In total, 1632 PLWH were tested for SARS-CoV-2 infection among the 20,662 who received care within the MedStar healthcare system, Figure 1. Characteristics of PLWH who tested for SARS-CoV-2 versus those who did not test for SARS-CoV-2 are outlined in Table S1. A total of 249 PLWH had a SARS-CoV-2 PCR confirmed infection, and an additional 32 had an ICD-10 coded COVID-19 diagnosis for a total of 281 PLWH with COVID-19.

The mean age of PLWH with COVID-19 was 51.5 (SD 12.7) years, 63% (*n* = 177) were male, and 86% (*n* = 237) were African American/Black. The median CD4 + T lymphocyte count was 623 cells/mm^3^ [IQR 383, 938] and 87% had a HIV viral load <200 copies/mL. Characteristics of tested PLWH with COVID-19 versus without COVID-19 are described in Table S2. Among those tested for SARS-CoV-2, PLWH who tested positive had lower rates of chronic renal disease (11% versus 12%, *p* = 0.030), higher rates of obesity (40% versus 33, *p* = 0.046), and higher median nadir CD4 + T lymphocyte counts (533 versus 413, *p* = 0.036). Among those tested, mean age in years, sex at birth, healthcare insurance status, CD4+ T lymphocyte count, and total number with HIV viral load <200 copies/mL were similar between those with and without SARS-CoV-2 infection.

**Figure 2.**
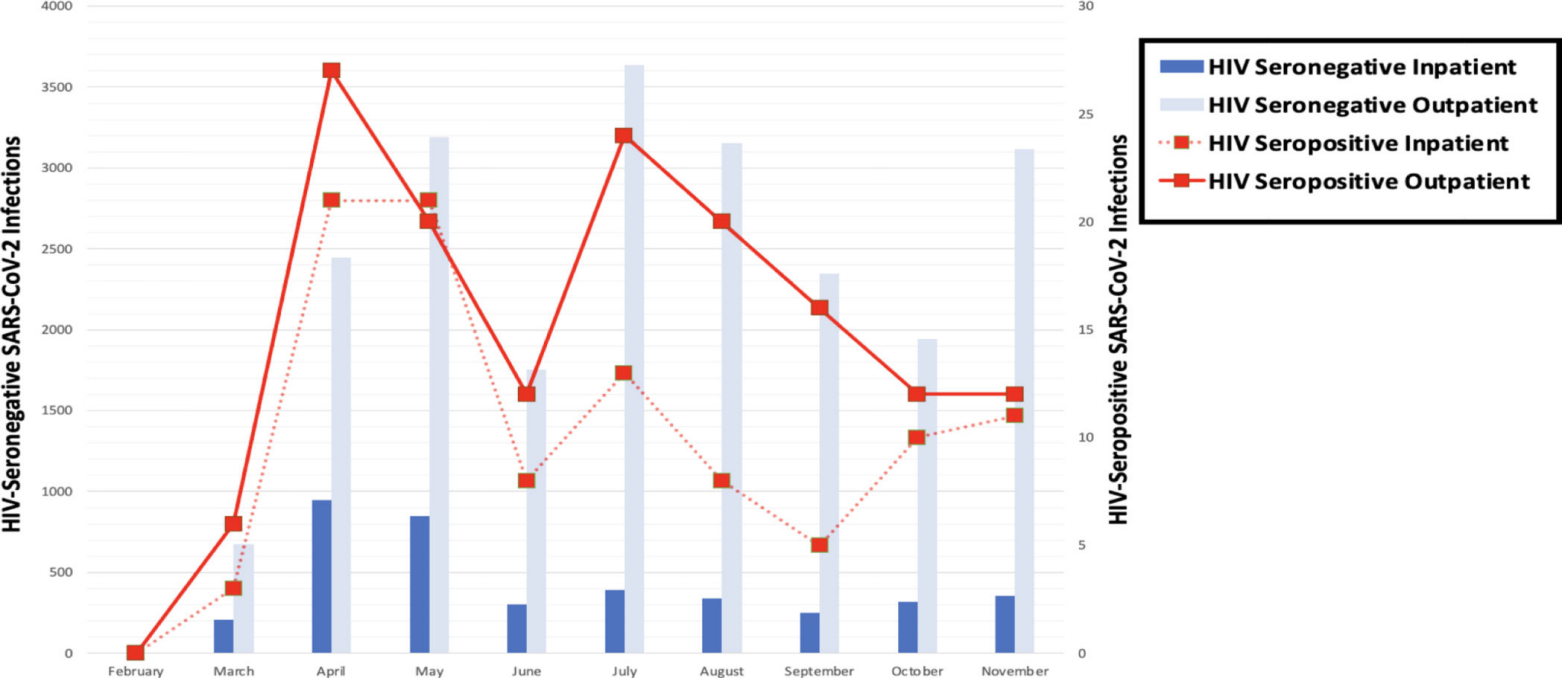
Persons with COVID-19 seen in the MedStar Healthcare system. Figure of persons living with HIV and HIV-seronegative individuals seen in the MedStar Healthcare system with COVID-19. Abbreviations: HIV, Human Immunodeficiency Virus; COVID-19, coronavirus disease 2019.

Incident inpatient and outpatient SARS-CoV-2 infections among PLWH and HIV-seronegative individuals are depicted in Figure 2. Compared to age- and sex-matched HIV-negative individuals, more PLWH were hospitalized at 47% (*n* = 132) versus 24% (*n* = 269), *p* < 0.001. Characteristics of PLWH and the matched cohort of HIV-seronegative individuals with COVID-19 are outlined in Table 1. The majority of PLWH, 86% (*n* = 237) were Black/African American versus 44% (*n* = 388) in the matched HIV-negative control group, *p* < 0.001. There were more privately insured persons in the HIV-negative group at 75% (*n* = 818) versus 58% (*n* = 161), *p* < 0001 among PLWH. Comorbid conditions were more common among PLWH including chronic liver disease at 24% (*n* = 70) versus 6% (*n* = 62), *p* < 0.001; hypertension 59% (*n* = 165) versus 36% (*n* = 403), *p* < 0.001; cardiovascular disease 62% (*n* = 174) versus 38% (*n* = 423), *p* < 0.001; malignancy 10% (*n* = 28) versus 2% (*n* = 19), *p* < 0.001; chronic lung disease 31% (*n* = 86) versus 12%(*n* = 134), *p* < 0.001; chronic renal disease 25% (*n* = 69) and 10% (*n* = 117), *p* < 0.001; and diabetes 33% (*n* = 92) versus 20% (*n* = 226), *p* < 0.001. However, rates of post-COVID-19 cardiovascular, thrombotic, AKI, and infection events were similar between HIV-seropositive and HIV-seronegative groups overall as well among individuals who required hospitalization.

Among those hospitalized, the PLWH had higher prevalence of liver disease at 16% (*n* = 21) versus 4% (*n* = 10), *p* < 0.001) and malignancy 19% (*n* = 25) versus 7% (*n* = 19), *p* = 0.001. The oxygen requirements and treatments were similar between the HIV-seropositive and HIV-seronegative groups. A total of 33% (*n* = 44) PLWH versus 31% (*n* = 83), *p* = 0.648, of HIV-seronegative individuals were admitted to the ICU. Among those admitted to the ICU, median length of stay was shorter among PLWH at 3 [IQR 1, 7.25] days versus 7 [IQR 3, 15] days. A total of 33% (*n* = 44) PLWH versus 31% (*n* = 83), *p* = 0.648, of HIV-seronegative individuals were admitted to the ICU. Inpatient mortality was similar for PLWH (*n* = 18/132) and HIV-seronegative individuals (*n* = 33/269) at 14% versus 13%, respectively, *p* = 0.750. The median length of hospital stay was 6 days [IQR 3, 11] for PLWH and 5.5 days [IQR 3, 11] for HIV-seronegative individuals, *p* = 0.889. Overall mortality was 6% (*n* = 18/281) in PLWH versus 3% (*n* = 33/1124) in HIV-seronegative individuals, *p* < 0.0001.

In analyses adjusted for age, sex, insurance status and multimorbidity, hospitalization among PLWH associated with older age aOR 1.04 (95% CI 1.01, 1.06), Medicaid insurance aOR 2.61 (95% CI 1.39, 4.97) and multimorbidity aOR 2.98 (95% CI 1.72, 5.23) (Table 2). Death/mechanical ventilation requirement was associated with older age aOR 1.06 (95% CI 1.01, 1.11), Medicaid insurance aOR 3.6 (95% CI 1.36, 9.74), and multimorbidity aOR 4.4 (95% CI 1.55, 15.9) in analyses adjusted for age, sex, insurance status and multimorbidity (Table 3).

## 4. Discussion

In a clinical cohort of PLWH seeking healthcare in the mid-Atlantic US, 281 were diagnosed with COVID-19 between March 2020 and November 2020. Patients were seen in a variety of care settings including ambulatory and inpatient. This group was predominantly African American/Black, carried a heavy burden of prevalent medical comorbidities, and had mostly virologically well controlled HIV. PLWH were admitted to the inpatient setting more frequently than an age- and sex-matched cohort and had a higher overall mortality rate, but once admitted, had similar mortality to their HIV-seronegative counterparts. Despite more frequent hospital admissions, more comorbid illnesses, and higher overall mortality COVID-19 associated complications including AKI, thrombosis, cardiovascular events, and other infections were similar between PLWH and the matched HIV-seronegative control group. As in the general population, older age and multimorbidity are associated with more severe outcomes. Differences in outcomes were also noted by insurance status, some of which may be attributed to age but may also reflect social determinants of health. This study adds to the existing studies that suggest that older age, multiple medical comorbidities, and social determinants of health influence on COVID-19 outcomes.

COVID-19-associated complications such as cardiovascular events, AKI, and thrombotic events are frequently noted in the literature. AKI frequently complicates SARS-CoV-2 infection, and incidence rates are variable, but rates of up to 57% are reported among those hospitalized and/or admitted to an intensive care unit [21] AKI in the setting of COVID-19 is associated with Black/African American or Hispanic race, male sex, older age and other comorbidities such as diabetes, cardiovascular disease, hypertension, or baseline chronic kidney disease [21–24]. Further, myocardial injury is commonly reported among hospitalized patient with COVID-19, with risk factors being older age and a history of cardiovascular disease [25]. Thrombotic events are also common in persons with COVID-19 and pulmonary emboli and deep venous thrombosis have been reported in 20 to 30% of persons with COVID-19 with risk factors of older age and cardiovascular disease [30,31,44]. Persons with deep venous thrombosis and COVID-19 were older, had higher rates of cardiac injury, and oxygenation index [45]. HIV is a known risk factor for chronic kidney disease, cardiovascular disease, and venous thrombosis [46–49]. PLWH also have known higher rates of medical comorbidities, which was reflected in our cohort [34–37]. Thus, we initially hypothesized higher rates of COVID-19 associated complications. Studies are limited regarding COVID-19-associated complications among PLWH, but Durstenfeld et al. reported that hospitalized PLWH did not have elevated risk of major adverse cardiac events, mortality, or severity of disease.^18^ Although the methodology was different, these findings, combined with our study findings of similar rates of COVID-19-related complications, suggest the need for additional study. Potential insights may be gained by further examining the immunologic response to disease among both PLWH as immune dysregulation is thought to contribute to SARS-CoV-2 pathogenesis including end organ disease such as myocardial injury, other cardiovascular dysfunction, or kidney dysfunction [50].

As other studies have reported, hospitalization rates were higher among PLWH in our cohort [14,51,52]. As more PLWH were admitted to the inpatient setting, overall mortality among all COVID-19 positive individuals, hospitalized and non-hospitalized was higher among PLWH versus the HIV-seronegative cohort. However, inpatient mortality was similar between PLWH and the HIV seronegative cohort. Differential hospitalization rates between the two groups may be reflective of differential admission practices and burden of comorbid disorders among PLWH. Our study corroborates the excess morbidity related to COVID-19 among PLWH found in other studies; however, our analysis may overestimate the mortality differences between PLWH and the HIV-seronegative group as we assume patients not admitted to our healthcare system survived infection and there may be unaccounted deaths in the outpatient group. Our study adds to the existing literature exploring the effect of COVID-19 on mortality among PLWH, yet additional work is needed to determine the effect of COVID-19 in PLWH as studies in various populations show differing outcomes [8,12,15,17,18,52–56].

We determined risk factors related to hospitalization for SARS-CoV-2 or death/mechanical ventilation and found they were associated with age, insurance status, and multimorbidity. These findings are similar to those noted in the general HIV-seronegative population where known risk factors for hospitalization or severe disease include older age, or the presence of other comorbid disorders [57–59]. This is corroborated in other studies of PLWH with COVID-19 older PLWH or those with multiple comorbidities have more severe disease/worse outcomes [19,20,54,60–62]. However, we did not observe more severe disease among ethnic/racial minorities as previously reported [14,60,63]. Our population was skewed with a majority of African American PLWH; thus, the assessment of the influence of race/ethnicity on COVID-19 severity is limited in our analysis. To our knowledge, the association of insurance type with hospitalization for COVID-19 or death/mechanical ventilation among PLWH has not been reported in the literature. However, those that are uninsured/self-pay and Medicaid recipients are socially and economically vulnerable populations [64,65], and the higher rates of severe COVID-19 disease may be reflective of differences in health seeking behavior, health service delivery, or other social determinants of health that are confounders in the relationship. The association of social determinants of health and COVID-19 outcomes has been described in the literature and access and delivery of care may be targets for intervention [66,67].

## 5. Limitations

Our study utilized the data available in the Medstar Health electronic medical registry, so we are unable to account for care sought outside this healthcare system, HIV treatment history, or duration of HIV infection. Additionally, all comorbid diagnoses were determined by ICD-10 codes. These codes were developed for administrative use and have their own inherent bias, but they are used by public health organizations to conduct surveillance and have also successfully been used by researchers including in large studies of COVID-19 [52,68–70]. To ensure the data accuracy we conducted manual abstraction of a subset of the population and the data accuracy was consistent with that of the published literature [41]. The majority of the participants in this study were virologically suppressed and sought healthcare. Thus, our findings may not be representative of persons with advanced HIV or not receiving care. Additionally, the therapeutic approaches to treatment and prevention of COVID-19 changed over the duration of the study which likely impacted outcomes and ability to compare our results with that of studies performed early in the pandemic. We utilized electronic health records that do not capture genomic surveillance data, but the data analyzed for this study included individuals diagnosed with COVID-19 between March 2020 and November 2020. The alpha (B.1.1.7.) variant was first reported in the United Kingdom in December of 2020. Thus, individuals in our cohort likely were likely infected with the original COVID-19 strain. The emergence of the alpha, delta, and omicron variants likely affected disease severity and outcomes and our findings may not be generalizable to those infected with other variants. Our cohort was predominantly African American/Black, and although representative of the HIV-epidemic in the area, this may have affected our outcomes as other studies have noted differences in outcomes by race. Other studies noted differences by race and COVID-19 outcomes, our population was predominantly African American and this may have influenced outcomes [71–73]. We were not able to fully assess difference by race given the unequal racial population distribution Additional studies are needed to identify emerging trends in hospitalizations, morbidity, and mortality among PLWH with more recent SARS-CoV-2 variants, and contemporary SARS-CoV-2 prevention and treatment modalities.

## 6. Conclusions

Our findings suggest disparities in COVID-19 morbidity among PLWH in the form of excess hospitalizations and higher mortality when including both inpatients and outpatient COVID-19 diagnoses. Despite higher burdens of baseline comorbid illness, PLWH did not experience more cardiovascular, acute kidney injury, or thrombotic events. Similar to other studies, older age, multimorbidity, and Medicaid insurance were associated with more severe outcomes among PLWH. Ongoing assessments of these findings and COVID-19 prevention efforts are needed among PLWH, especially socially or economically vulnerable populations, those with advanced age, or multiple comorbidities.

## Supplementary Material

Supplemental Tables 1

Supplemental Tables 2

## Figures and Tables

**Table 1.** Comparison of age and sex matched PLWH and age/sex matched HIV-seronegative individuals with COVID-19.

Characteristic	HIV-Seronegative (*n* = 1124)	HIV-Seropositive (*n* = 281)	*p*-Value
Age mean years (SD)	51.2 (13.7)	51.5 (12.7)	1
Sex at Birth, *n* (%)			
Male	708 (63)	177 (63)	1
Female	416 (37)	104 (37)	
Race, *n* (%)			
African American/Black	388 (44)	237 (86)	<0.001
White	341 (39)	20 (7)	
Other	157 (18)	18 (7)	
Ethnicity			
Non-Hispanic	813 (92)	258 (98)	0.001
Hispanic	69 (8)	6 (2)	
Insurance, *n* (%)			
Private	818 (75)	161 (58)	<0.001
Medicaid	79 (7)	67 (24)	
Medicare	146 (13)	45 (16)	
Non-Insured	46 (4)	6 (2)	
Co-Morbid Conditions, *n* (%)			
Cardiovascular Disease	423 (38)	174 (62)	<0.001
Hypertension	403 (36)	165 (59)	<0.001
Obesity	280 (25)	111 (40)	<0.001
Diabetes Mellitus	226 (20)	92 (33)	<0.001
Chronic Renal Disease	117 (10)	31 (11)	<0.001
Chronic Liver Disease	62 (6)	70 (25)	<0.001
Malignancy	19 (2)	28 (10)	<0.001
Transplant	14 (1)	8 (3)	0.062
Post-Infection Events, *n* (%)			
Thrombotic	2 (1)	1 (0.4)	0.489
Infections	14 (1)	7 (2)	0.206
Cardiovascular	22 (2)	9 (3)	0.253
Acute Kidney Injury	6 (1)	4 (1)	0.120
INPATIENT	N = 269	N = 132	
Median Length of Stay, days (IQR)	6 (3, 11)	5.5 (3, 11)	0.889
ICU Median Length of Stay, days (IQR)	7 (3, 15)	3 (1, 7.25)	0.008
Deceased, *n* (%)	33 (13)	18 (14)	0.750
Comorbid Conditions, *n* (%)			
Diabetes Mellitus	110 (41)	50 (38)	0.589
Cardiovascular Disease	67 (35)	35 (27)	0.716
Chronic Renal Disease	30 (11)	22 (17)	0.154
Chronic Liver Disease	10 (4)	21 (16)	<0.001
Malignancy	19 (7)	25 (18)	<0.001
Post-Infection Events, *n* (%)			
Thrombotic	1 (0.4)	1 (0.4)	0.551
Infections	12 (5)	3 (2)	0.421
Cardiovascular	20 (7)	5 (4)	0.190
Acute Kidney Injury	4 (2)	1 (1)	1
COVID Treatments ^a^, *n* (%)			
Remdesivir	39 (15)	18 (14)	0.880
Dexamethasone	65 (24)	32 (24)	1
Azithromycin	120 (45)	58 (44)	0.915
Hydroxychloroquine	49 (18)	22 (17)	0.781
Tocilizumab	18 (7)	6 (5)	0.504
Supplemental Oxygen, *n* (%)			
Room Air	38 (18)	27 (25)	
Nasal Cannula	82 (39)	33 (30)	0.097
Non-Rebreather/HFNC	29 (14)	25 (23)	
Ventilator	53 (25)	21 (19)	
Laboratory Data (Admission ^b^), (IQR)			
Median WBC (×10^3^ cells/μL)	7 (5.3, 9.6)	6.80 (4.60, 9.20)	0.060
Median Absolute Lymphocyte count (×10^3^ cells/μL)	1.05 (0.80, 1.50)	1.30 (0.80, 1.80)	0.032
Mean Hemoglobin (gm/dL)	12.98 (12.98)	12.23 (2.40)	0.006
Mean Platelets (×10^3^ cells/μL)	233 (95.52)	211.07 (91.78)	0.036
Median Creatinine (mg/dL)	1.11 (0.83, 1.71)	1.17 (0.89, 2.48)	0.175
Mean eGFR (mL/min/1.73 m^2^)	50.07 (17.96)	46.10 (20.67)	0.062
Median ALT (IU/L)	37.00 (23, 58.50)	33.00 (22, 49.50)	0.224
Median CPK (units/L)	148.50 (78.25, 326)	142 (75, 394)	0.834
Median Troponin (ng/mL)	0.02 (0.01, 0.03)	0.02 (0.01, 0.04)	0.248
Median Procalcitonin (ng/mL)	0.19 (0.10, 0.73)	0.34 (0.10, 0.86)	0.398
Median Ferritin (ng/mL)	592 (300, 1330.40)	565.15 (262.40, 1367.22)	0.573
Median Lactate Dehydrogenase (units/L)	343.50 (264.50, 460.50)	312.50 (238.5, 161.5)	0.477
Median D-Dimer (mcg/mL FEU)	1.66 (0.78, 3.06)	1.44 (0.78, 3.46)	0.902
Median C-Reactive Protein (mg/L)	82.80 (35.05, 127.50)	93.70 (53.50, 161.50)	0.285
Laboratory Data (Peak), (IQR)			
Median WBC (×10^3^ cells/μL)	10.20 (6.93, 14.97)	8.90 (6, 12.70)	0.010
Median Platelets (×10^3^ cells/μL)	307 (236.75, 417.75)	263 (194, 371)	0.008
Median Procalcitonin (ng/mL)	0.22 (0.10, 2.30)	0.39 (0.10, 1.70)	0.498
Median Ferritin (ng/mL)	896.1 (373.45, 1817.15)	707.40 (345, 1798.20)	0.420
Median Lactate Dehydrogenase (units/L)	400.50 (284.50, 574)	355.5 (271, 576.75)	0.615
Median D-Dimer (mcg/mL FEU)	1.88 (1.09, 5.07)	1.94 (0.93, 3.83)	0.655
Mean C-Reactive Protein (mg/L)	109 (47.40)	121.50 (61.70, 172.25)	0.573
Median Interleukin-6 (pg/mL)	12.10 (5, 43.50)	9 (5, 18.60)	0.412

Abbreviations: PLWH, persons living with HIV; N, number; SD, standard deviation; IQR, interquartile range; HFNC, high-flow nasal cannula; WBC, white blood cell count; mcL, microliters; gm, gram; dL, deciliter; eGFR, estimated glomerular filtration rate; mg, milligrams; mL, milliliter; min, minute; m, meter; IU, CPK, creatinine phosphokinase; international units, L, liter; ng, nanogram; mcg, micrograms; pg, picogramss.

a Other investigational treatments/treatments included vagepant (*n* = 0, HIV-seronegative; *n* = 1, HIV-seropositive) and extracorporeal membrane oxygenation (ECMO) (*n* = 0, HIV-seronegative; *n* = 1, HIV-seropositive).

b Laboratory data are from admission or first available.

**Table 2.** Factors associated with hospitalization among persons living with HIV with COVID-19.

Characteristic	OR	*p*-Value	aOR *	*p*-Value
Age, years	1.05 (1.03, 1.07)	<0.001	1.04 (1.01, 1.06)	0.002
Sex at Birth				
Female (reference)	-	-	-	-
Male	1.12 (0.69, 1.83)	0.646	1.5 (0.87, 2.62)	0.152
Race				
White (reference)	−	−	−	−
African American/Black	0.6 (0.23, 1.5)	0.278	−	−
Other	0.26 (0.06, 0.96)	0.051	−	−
Ethnicity				
Non-Hispanic (reference)	−	−	−	−
Hispanic	1.13 (0.21, 6.22)	0.88	−	−
HIV Viral Load				
<200 (reference)	−	−	−	−
>200	1 (1, 1)	0.34		
CD4 + T Lymphocyte				
>200 (reference)	−	−	−	−
<200	0.98 (0.91, 1.06)	0.624	−	−
Insurance				
Private (reference)	−	−	−	−
Medicaid	2.22 (1.25, 4)	0.007	2.61 (1.39, 4.97)	0.003
Medicare	2.4 (1.23, 4.77)	0.011	1.41 (0.67, 3.01)	0.362
Uninsured/Self-Pay	0.8 (0.11, 4.22)	0.798	0.78 (0.09, 5.39)	0.803
Multimorbidity **	3.74 (2.29, 6.21)	<0.001	2.98 (1.72, 5.23)	<0.001

Abbreviations: SARS CoV-2, severe acute respiratory syndrome coronavirus 2; OR, odds ratio; aOR, adjusted odds ratio.

* Multivariable model adjusted for age, sex, insurance status, and multimorbidity,

** Multimorbidity = two or more comorbidities including cardiovascular disease, obesity, diabetes mellitus, chronic renal disease, or malignancy.

**Table 3.** Factors associated with death/mechanical ventilation among hospitalized PLWH with COVID-19.

Characteristic	OR (CI 95%)	*p*-Value	aOR *	*p*-Value

Age, years	1.06 (1.03, 1.1)	<0.0001	1.06 (1.01, 1.11)	0.013
Sex at Birth				
Female (reference)	−	−	−	−
Male	0.87 (0.4, 1.93)	0.72	0.97 (0.42, 2.34)	0.94
Race				
White (reference)	−	−	−	−
African American/Black	2.34 (0.46, 42.92)	0.416	−	−
Other	0 (0, 2.44 × 10^15^)	0.987	−	−
Ethnicity				
Non-Hispanic (reference)	−	−	−	−
Hispanic	1.95 (0.1, 12.76)	0.55	−	−
HIV Viral Load				
<200 (reference)	−	−	−	−
>200	0.97 (0.84, 1)	0.592	−	−
CD4 + T Lymphocyte				
>200 (reference)	−	−	−	−
<200	1.05 (0.86, 1.31)	0.64	−	−
Insurance				
Private (reference)	−	−	−	−
Medicaid	2.97 (1.19, 7.49)	0.019	3.6 (1.36, 9.74)	0.01
Medicare	2.78 (0.95, 7.73)	0.051	1.33 (0.42, 3.99)	0.614
Uninsured/Self-Pay	7.55 (0.97, 43.98)	0.029	12.09 (1.17, 1.26 × 10^2^)	0.027
Multimorbidity **	6.55 (2.47, 22.68)	<0.001	4.4 (1.55, 15.9)	0.011

Abbreviations: OR, odds ratio; aOR, adjusted odds ratio.

* Multivariable model adjusted for age, sex, insurance status, and multimorbidity

** Multimorbidity = two or more comorbidities including cardiovascular disease, obesity, diabetes mellitus, chronic renal disease, or malignancy.

## Data Availability

Data is contained within the article and Supplement Material.
