# Supplementary material for: COVID-19 Outcomes in a US Cohort of Persons Living with HIV (PLWH)
Source: Reports (MDPI). Author manuscript; Available in PMC 2023 Apr 14. (PMC10104440; doi:10.3390/reports5040041)
Supplement: Supplemental Tables 1 [file NIHMS1871544-supplement-Supplemental_Tables_1.pdf]

SUPPLEMENTAL TABLE S1: Characteristics of PLWH and SARS-CoV-2 testing status

	All (n= 20662)	SARS-CoV-2 Tested (NO) (n=19030)	SARS-CoV-2 Tested (YES) (n=1632)
Mean Age, years (SD)	51.9 (13.8)	51.9 (13.8)	51.2 (13.4)
Sex at Birth, n (%)			
Male	13296 (79)	12336 (65)	960 (59)
Female	7363 (36)	6691 (26)	672 (41)
Race, n (%)			
African-American/Black	15592 (79)	14265 (78)	1327 (83)
White	3262 (16)	3065 (17)	197 (12)
Other	976 (5)	900 (5)	76 (5)
Ethnicity, n (%)			
Non-Hispanic	18945 (98)	17402 (98)	1543 (99)
Hispanic	476 (3)	459 (3)	17 (1)
Insurance, n (%)			
Private	10580 (51)	9713 (51)	867 (53)
Medicaid	5555 (27)	5138 (27)	417 (26)
Medicare	3431 (17)	3115 (16)	316 (19)
Non-Insured	1000 (5)	974 (5)	26 (2)
Median CD4+ T Lymphocytes (cells/mm^3) [IQR]	621 [406, 859]	626 [419, 862]	564 [347, 816]
HIV Viral Load copies/mL <200, n (%)	1745 (90%)	1494 (91%)	251 (87%)

Abbreviations: PLWH, persons living with HIV; SARS CoV-2, severe acute respiratory syndrome coronavirus 2; N, number; SD, standard deviation; IQR, interquartile range; mL, milliliter
