# Supplementary material for: COVID-19 Outcomes in a US Cohort of Persons Living with HIV (PLWH)
Source: Reports (MDPI). Author manuscript; Available in PMC 2023 Apr 14. (PMC10104440; doi:10.3390/reports5040041)
Supplement: Supplemental Tables 2 [file NIHMS1871544-supplement-Supplemental_Tables_2.pdf]

SUPPLEMENTAL TABLE S2: Characteristics of persons living with HIV who completed SARS-CoV-2 testing with and without infection

Characteristic	(NO) SARS-CoV-2 (n=1351)	(YES) SARS-CoV-2 (n=281)	*p-value*
Age mean years (SD)	51.2 (13.6)	51.5 (12.74)	0.698
Sex at Birth, n (%)			
Male	783 (58)	177 (63)	0.135
Female	568 (42)	104 (37)	
Race, n (%)			
African-American/Black	1090 (82)	237 (86)	0.009
White	177 (13)	20 (7)	
Other	58 (4)	18 (7)	
Ethnicity, n (%)			
Non-Hispanic	1285 (99)	258 (98)	0.088
Hispanic	11 (1)	6 (2)	
Insurance, n (%)			
Private	706 (52)	161 (58)	0.251
Medicaid	350 (26)	67 (24)	
Medicare	271 (20)	45 (16)	
Non-Insured	20 (2)	6 (2)	
Median CD4 + T Lymphocytes cells/mm^3 n, [IQR][a]	552 [310,798]	623 [383, 938]	0.159
Median Nadir CD4 + T Lymphocytes cells/mm^3 n, [IQR][b]	413 [219, 654]	533[314, 724]	0.036
HIV Viral Load copies/mL <200, n (%)[c]	201(87)	50 (87)	1
Current HIV Treatments, n (%)			
INSTI	716 (52)	148 (53)	0.972
PI	432 (32)	77 (27)	0.151
NNRTI	312 (23)	74 (26)	0.278
NRTI	1060 (79)	227 (81)	0.431
Co-Morbid Conditions, n (%)			
Cardiovascular Disease	877 (65)	174 (62)	0.376
Hypertension	827 (61)	165 (59)	0.476
Obesity	447 (33)	111 (40)	0.046
Chronic Liver Disease	407 (30)	70 (25)	0.094
Diabetes Mellitus	386 (29)	92 (33)	0.185
Chronic Renal Disease	423 (31)	69 (25)	0.030
Malignancy	123 (9)	28 (10)	0.734
Transplant	50 (3)	8 (3)	0.599

Abbreviations: PLWH, persons living with HIV; N, number; SD, standard deviation; IQR, interquartile range; mL, milliliter; INSTI, integrase inhibitor; PI, protease inhibitor; NNRTI, non-nucleoside reverse transcriptase inhibitors; NRTI, nucleoside reverse transcriptase inhibitor

[a]Where available the most recent CD4+ T lymphocyte count within one year prior to the SARS-CoV-2 infection was abstracted.

[b]The lowest ever CD4+ T lymphocyte count available in the MedStar database was abstracted.

[c]Where available the most recent HIV viral load within one year prior to the SARS-CoV-2 infection was abstracted
